# Supplementary figures and images for: Frequency of Androgen Receptor Positivity in Tumors: A Study Evaluating More Than 18,000 Tumors
Source: Biomedicines. 2024 Apr 25;12(5):957. doi: 10.3390/biomedicines12050957 (PMC11117763; doi:10.3390/biomedicines12050957)

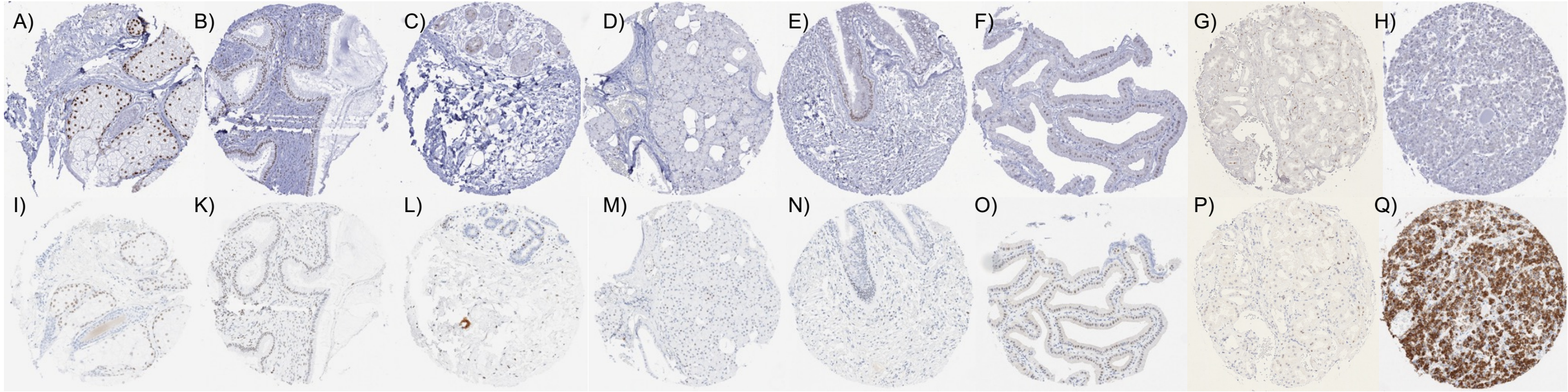

Supplement: Supplementary file 1 [file biomedicines-12-00957-s001.zip › Figure S1.pdf]

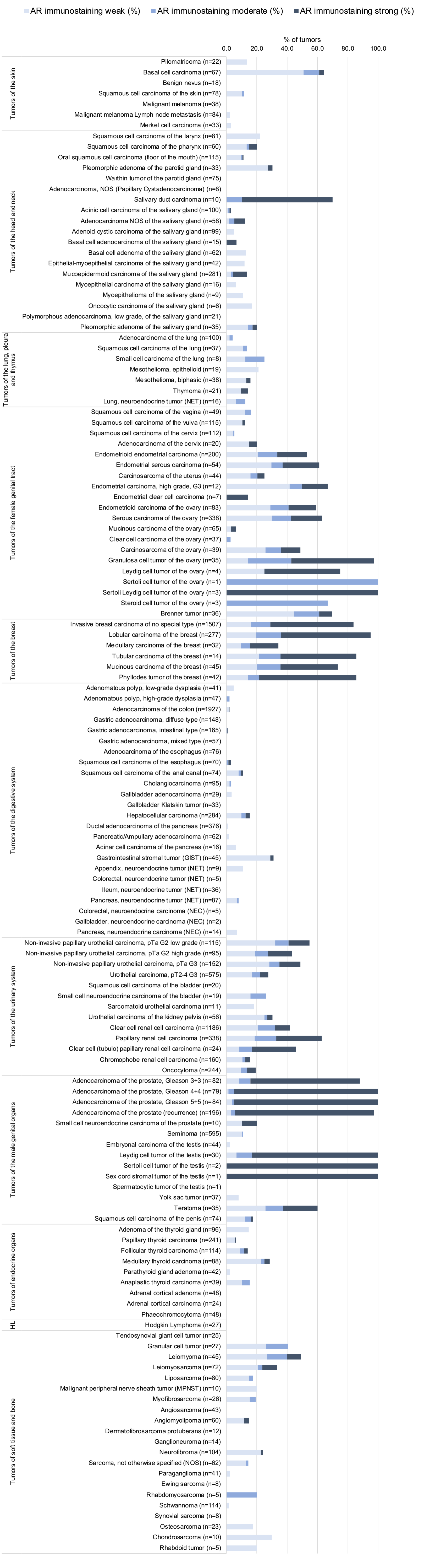

Supplement: Supplementary file 1 [file biomedicines-12-00957-s001.zip › Figure S2.pdf]

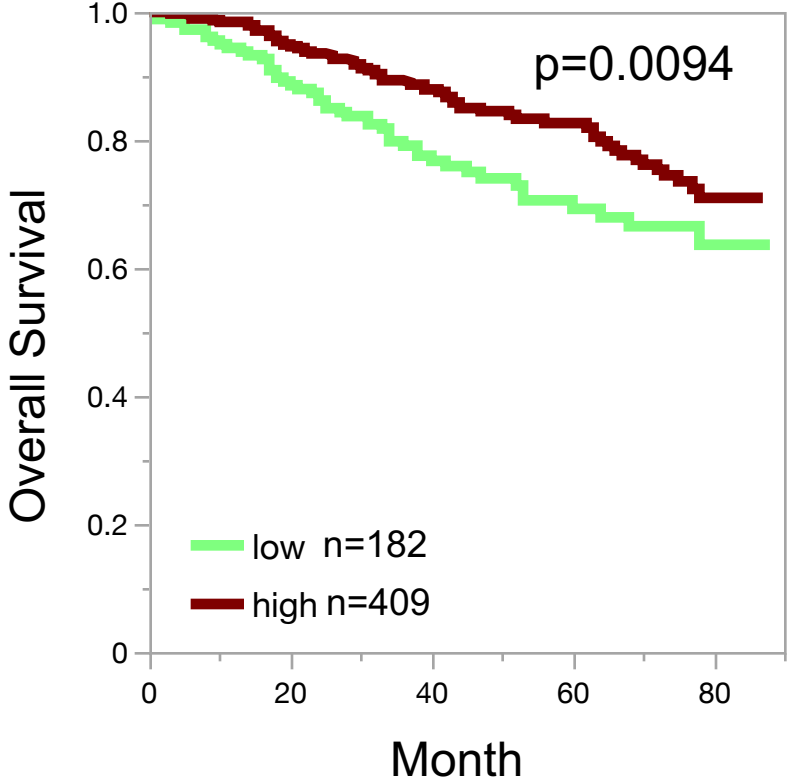

Supplement: Supplementary file 1 [file biomedicines-12-00957-s001.zip › Figure S3.pdf]
